# The *Superoxide dismutase* (*SOD*) Gene Family in Litchi (*Litchi chinensis* Sonn.): Identification, Classification, and Expression Responses in Leaves Under Abiotic Stresses

**DOI:** 10.3390/antiox15010014

**Published:** 2025-12-22

**Authors:** Chao Fan, Jie Yang, Rong Chen, Wei Liu

**Affiliations:** Guangdong Provincial Key Laboratory of Science and Technology Research on Fruit Tree, Key Laboratory of South Subtropical Fruit Biology and Genetic Resource Utilization, Ministry of Agriculture and Rural Affairs, Institute of Fruit Tree Research, Guangdong Academy of Agricultural Sciences, Guangzhou 510640, China; yangjie95222@126.com (J.Y.); rongchen9821@126.com (R.C.); liuwei1@gdaas.cn (W.L.)

**Keywords:** litchi, *SOD* genes, evolution, expression pattern, abiotic stress

## Abstract

Superoxide dismutase (SOD) serves as a critical enzyme that is involved in plant development and abiotic stresses by effectively detoxifying reactive oxygen species (ROS). Though the *SOD* gene family has been reported across various plant species, its specific members and functional roles in litchi (*Litchi chinensis* Sonn.) remain poorly understood. In this study, a total of seven *SOD* (christened *LcSOD*) genes were identified from the litchi genome and classified into three groups across six chromosomes. Notably, genes from the same evolutionary branch had more similar structures and motif distributions. The *LcSOD* genes were confirmed to have a stronger collinearity with dicotyledons than with monocotyledons. Cis-acting elements analysis indicated that the *LcSOD* gene family was deeply involved in orchestrating growth, development, and responses to multiple phytohormones and diverse stresses. Expression patterns of the *LcSOD* genes across different tissues revealed universal and specific expressions. In leaves, expression levels of the *LcSOD* genes were induced by cold, heat, drought, and salt stresses, and transcript levels correlated positively with concomitant changes in key physiological parameters under the same conditions. In addition, the *LcSOD* genes were characterized for their physicochemical properties, subcellular localizations, secondary and tertiary structures, gene ontology (GO) annotations, and protein-protein interactions. Our findings offer comprehensive insights into the *LcSOD* gene family, enriching genetic resources. They provide a framework for functional characterization and the development of stress-resistant cultivars, driving both basic research and applied breeding programs in litchi.

## 1. Introduction

As sessile organisms, plants are more vulnerable to various environmental stresses, which can significantly impede their growth and development [1]. When exposed to stresses, plants are bound to accumulate reactive oxygen species (ROS). Moderate levels of ROS function as signaling molecules to enhance plant stress resistance by triggering adaptive protective mechanisms. Nevertheless, excessive ROS accumulation drives cellular membrane lipid peroxidation, triggering irreversible metabolic damage and compromising cellular integrity, ultimately leading to cell death [2,3]. Plants have constructed a variety of enzymatic and non-enzymatic defense systems to combat ROS accumulation. Among these enzymatic defense systems, SOD is a ubiquitous metalloprotein recognized as the first line of defense against oxidative damage in plant cells. It catalyzes the dismutation of superoxide radicals (O_2_^−^) into hydrogen peroxide (H_2_O_2_) and oxygen (O_2_), regulating intracellular ROS homeostasis [4,5].

SODs fall into four distinct classes based on their metal cofactors, including copper/zinc SOD (Cu/Zn-SOD), manganese SOD (Mn-SOD), iron SOD (Fe-SOD), and nickel SOD (Ni-SOD) [6]. Cu/Zn-SOD is mainly present in higher plants, Mn-SOD and Fe-SOD are mainly present in non-vascular plants, and Ni-SOD is predominantly found in *Streptomyces*, cyanobacteria, and a few green algae [7,8]. These SODs are encoded by nuclear genes that determine their localizations in different cellular compartments. Cu/Zn-SODs are chiefly located in mitochondria, chloroplasts, and cytosol, whereas Mn-SODs are found in mitochondria and peroxisomes. By contrast, Fe-SODs are chiefly situated in mitochondria, chloroplasts, and peroxisomes [9]. Mn-SOD and Fe-SOD share high sequence homology, which may have evolved from the same ancestral enzyme, whereas Cu/Zn-SOD shows no significant sequence similarity [10].

The *SOD* gene family and its responses to abiotic stresses have been extensively identified and functionally characterized in numerous plant species. In Arabidopsis (*Arabidopsis thaliana* (L.) Heynh.) rosette leaves, *AtCSD1* and *AtCSD2* exhibited significant elevation within 60–125 μmol·m^−2^·s^−1^·PAR, whereas *AtCSD3* showed a significant increase exclusively in 500 μmol·m^−2^·s^−1^·PAR; *AtFSD1* decreased within the initial 4 h of exposure to fumigation and rebounded to pre-treatment baselines between 48 and 72 h post-fumigation [11]. In rice (*Oryza sativa* L.), *OsMSD1* overexpression up-regulated ROS scavenging, chaperone, and quality control systems to enhance heat tolerance; conversely, *OsMSD1*-knock-down rice exhibited notable susceptibility to heat [12]. In banana (*Musa acuminata* Colla.) leaves, *MaCSD2A* and *MaCSD1C* were up-regulated, whereas *MaCSD1D* and *MaCSD2B* were markedly down-regulated under cold stress; as for heat stress, *MaCSD1B* and *MaCSD1D* showed increasing transcription levels, while *MaCSD2A* was down-regulated after 12 h [13]. In wheat (*Triticum turgidum* L. subsp. *Durum*), *TdMnSOD* was induced by salt, drought, and H_2_O_2_, and its overexpression enhanced tolerance to different abiotic stresses in recombinant yeast cells and transgenic Arabidopsis plants [14]. In Liriodendron (*Liriodendron chinense* (Hemsl.) Sarg.) leaves, two *LchSOD* genes rapidly increased and peaked at 1 h post-heat; eight *LchSOD* genes peaked at 3 h post-drought; and six *LchSOD* genes reached their peak at 12 h post-cold [15]. In herbaceous peony (*Paeonia lactiflora* Pall.) leaves, seven *PlSOD* genes underwent significant upregulation under heat stress [16]. These findings highlight the versatility and importance of *SOD* genes in enabling plants to resist various environmental challenges.

Litchi (*Litchi chinensis* Sonn.), a subtropical fruit tree native to South China, is widely cultivated for its nutritious and delicious fruits, which possess significant economic and cultural importance [17]. However, the cultivation of this species is impeded by significant challenges, primarily attributed to its pronounced sensitivity to abiotic stresses such as drought, salt, and extreme temperatures. These factors not only compromise fruit yield and quality but also impose restrictions on the feasible cultivation zones of litchi [18]. It is against this backdrop that the identification and functional analysis of the *LcSOD* gene family are crucial. A current database [19] was utilized in this study to conduct genome-wide identification and characterization of the *LcSOD* gene family members, including protein properties, evolutionary relationship, structural characteristics, conserved motifs, chromosomal localizations, collinear inspections, gene ontology (GO) enrichment, protein interaction network, cis-acting elements, and organizational expression. Finally, the *LcSOD* gene expression profiles and physiological data from litchi leaves under several abiotic stresses (cold, heat, drought, and salt) were detected and compared. Our research systematically characterized the *LcSOD* gene family and elucidated its expression responses to cold, heat, drought, and salt stresses, providing a molecular foundation for breeding stress-resilient cultivars of litchi.

## 2. Materials and Methods

### 2.1. Plant Materials and Treatments

Annual seedlings of ‘Feizixiao’ litchi (known for its widespread cultivation, high yield, and strong adaptability in China) were cultivated in a greenhouse at the Institute of Fruit Tree Research, Guangdong Academy of Agricultural Sciences (113°22′41.200″ E, 23°9′32.418″ N) using growth substrate consisting of a mixture of sandy red soil, peat soil, and coconut bran silk with a volume ratio of 3:1:1 and pH values ranging from 5.5 to 6.5. After plants had developed twenty-five true leaves with robust growth, they were placed to stress treatments (4.0 ± 1.0 °C, 38.0 ± 0.5 °C, 20% (*w*/*v*) PEG6000, and 400 mmol/L NaCl) selected based on environmental extremes experienced by litchi in subtropical production regions [18]. Samples of upper young leaves were collected after 0, 3, 6, 12, and 24 h of each treatment, with three biological replicates per time point. These samples underwent rapid freezing via liquid nitrogen and were preserved at −80 °C for later use.

### 2.2. Identification and Bioinformatic Analysis of the LcSOD Gene Family

Hidden Markov Model (HMM) profiles of SOD domains (PF00080, PF00081, and PF02777) were obtained from the InterPro database (https://www.ebi.ac.uk/interpro/, accessed on 22 July 2024). The LcSOD protein sequences were searched in the litchi genome database using the aforementioned HMM profiles with HMMER 3.0, setting an E-value < 1 × 10^−5^. Sequences that lacked SOD domains were further eliminated using the CDD (https://www.ncbi.nlm.nih.gov/cdd/?term=, accessed on 22 July 2024), InterPro, and SMART (http://smart.embl-heidelberg.de/, accessed on 22 July 2024) databases. Physicochemical properties of the LcSOD proteins, including sequence length, molecular mass, theoretical isoelectric point (pI), instability index (II), and grand average of hydropathicity (GRAVY), as well as their transmembrane domains, subcellular locations, and secondary structures were analyzed using Expasy-ProtParam (https://web.expasy.org/protparam/, accessed on 28 July 2024), TMHMM-2.0 (https://services.healthtech.dtu.dk/services/TMHMM-2.0/, accessed on 6 August 2024), CELLO v2.5 (https://cello.life.nctu.edu.tw/, accessed on 10 August 2024), and SOPMA (https://npsa-prabi.ibcp.fr/cgi-bin/npsa_automat.pl?page=npsa_sopma.html, accessed on 15 August 2024) predictors, respectively.

Amino acid sequences of *SOD* genes derived from Arabidopsis, rice, and litchi (Table S1) were subjected to phylogenetic analysis using the neighbor-joining (NJ) method in MEGA 7.0 software, with a bootstrap value of 1000 and other parameters set as default. Chromosomal locations of the *LcSOD* genes were ascertained by employing a Gene Location Visualization from the GTFIGFF plugin in TBtools-II V2.326 software [20], referencing litchi genome annotation file. Collinear graphs of *SOD* genes were constructed by collectively utilizing One Step MCScanX and Advanced Circos plugins from TBtools-II V2.326, comparing litchi against litchi, Arabidopsis, longan (*Dimocarpus longan* Lour.), rice, and pineapple (*Ananas comosus* (L.) Merr.).

Intron/exon structures of the *LcSOD* genes were performed using GSDS 2.0 server (https://gsds.gao-lab.org/, accessed on 22 August 2024). Up to ten motifs of the LcSOD proteins were examined using by MEME server (https://meme-suite.org/meme/tools/meme, accessed on 30 August 2024), and conserved motifs were subsequently draw using a Visualize MEME/MAST Motif Pattern plugin from TBtools-II V2.326.

GO annotations of the *LcSOD* genes were analyzed using EGGNOG-MAPPER server (http://eggnog-mapper.embl.de/, accessed on 10 September 2024) and visualized via Go Enrichment and Enrichment Bar Plot plugins from TBtools-II V2.326. An interaction network between the LcSOD proteins was constructed via STRING predictor (https://cn.string-db.org/, accessed on 20 September 2024) using Arabidopsis as the reference species and requiring a minimum interaction score of 0.40, after which the visualization was performed using Cytoscape 3.10.3 software.

2000 bp upstream sequences from the starting codon of the *LcSOD* genes were submitted to PlantCARE sever (http://bioinformatics.psb.ugent.be/webtools/plantcare/html/, accessed on 25 September 2024) for cis-acting element prediction, and the results were then visualized using a HeatMap plugin from TBtools-II V2.326. Expression data of the *LcSOD* genes from roots, leaves, male flowers, female flowers, ovaries, carpopodiums, pericarp, fruitlets, aril, and seeds were obtained from litchi genome database and depicted via a HeatMap plugin using FPKM normalization.

### 2.3. Expression Patterns of the LcSOD Gene Family in Response to Abiotic Stresses

Total RNA was extracted from leaf samples using a Plant Total RNA kit containing DNase I (SIMGEN, Hangzhou, China), and its quality was subsequently assessed using a NanoDrop-2000c-type micro-spectrophotometer (Thermo Fisher Scientific, Guangzhou, China). First-strand cDNAs were synthesized using a cDNA First Strand Synthesis kit (SIMGEN, Hangzhou, China). Quantitative real-time PCR (qRT-PCR) experiments were performed using a 2 × SYBR Green PCR Mix kit (SIMGEN, Hangzhou, China) on a QuantStudioTM 3 Real-Time PCR system (Thermo Fisher Scientific, Guangzhou, China). Expression levels of the *LcSOD* genes were calculated using the 2^−ΔΔCt^ method [21]. The *LcActin* gene [22] was selected as the internal reference gene, and the primers used in this study are presented in Table S2.

### 2.4. Physiological Parameters of Litchi Leaves in Response to Abiotic Stresses

Activities of peroxidase (POD), catalase (CAT), and SOD, as well as contents of malondialdehyde (MDA), proline (PRO), soluble protein (SP), hydrogen peroxide (H_2_O_2_), and soluble sugar (SS), were measured using corresponding kits (Boxbio Science & Technology, Beijing, China) on a Spark^®^ 20M multimode microplate reader (Technical Analysis Equipment, Salzburg, Austria). Assays were conducted following kit manufacturer instructions.

Concretely, leaf tissue (0.1 g) was homogenized in 1 mL ice-cold extraction buffer (provided in each kit) at 1:10 (*w*/*v*), followed by centrifugation at 4 °C and 8000× *g* for 10 min, and supernatants were collected for all assays except PRO and SS. For PRO assay, the extraction buffer was boiled for 10 min post-homogenization before centrifugation at 10,000× *g*. For SS assay, samples were incubated at 95 °C for 10 min before centrifugation at 8000× *g*. All supernatants were collected for subsequent analyses. POD activity was determined by guaiacol oxidation at 470 nm (30–90 s interval): 150 μL reagent one, 20 μL reagent two, 20 μL reagent three, and 10 μL crude enzyme extract. CAT activity was determined by ultraviolet absorption at 240 nm (5–65 s interval): 190 μL working fluid and 10 μL crude enzyme extract. SOD activity was determined by nitro-blue tetrazolium reduction at 560 nm: 90 μL reagent one, 10 μL reagent two, 40 μL reagent three, 40 μL reagent five, and 20 μL crude enzyme extract. MDA content was determined by thiobarbituric acid reaction at 450, 532, and 600 nm: 50 μL reagent one, 250 μL working fluid, 100 μL reagent three, and 150 μL crude enzyme extract. PRO content was determined by acidic-ninhydrin at 520 nm: 200 μL acetic acid, 200 μL chromogen, 400 μL methylbenzene, and 200 μL crude enzyme extract. SP content was determined by Bradford at 595 nm: 1000 μL 1 × G-250 dye solution and 100 μL crude enzyme extract. H_2_O_2_ content was determined by titanium sulfate at 415 nm: 30 μL reagent two, 60 μL reagent three, 300 μL reagent four, and 300 μL crude enzyme extract. SS content was determined by anthrone-sulfuric acid at 620 nm: 40 μL distilled water, 20 μL reagent one, 200 μL sulfuric acid, and 40 μL crude enzyme extract.

### 2.5. Statistical Analysis

All experimental data are presented as the means ± standard errors of three technical replicates. Significance analysis of the data was conducted using one-way ANOVA with LSD multiple comparison test in DPS 9.01 software at a threshold of *p* < 0.05, and the results were then visualized using SigmaPlot 14.0 software. Correlation analysis between the *LcSOD* gene expression levels and physiological parameters of litchi leaves under abiotic stresses was performed using a Correlation Plot plugin from Origin 2024 software.

## 3. Results

### 3.1. Identification and Characterization of the LcSOD Genes

A total of seven *LcSOD* genes were obtained, designated as *LcSOD1* to *LcSOD7* according to their chromosomal localizations, and their protein sequences were analyzed. The results showed that the coding sequences ranged from 405 bp (LcSOD3) to 1194 bp (LcSOD1), the amino acids spanned from 134 aa (LcSOD3) to 397 aa (LcSOD1), the molecular masses varied from 13.73 kDa (LcSOD3) to 44.45 kDa (LcSOD1), the theoretical pI values reached from 4.86 (LcSOD5) to 9.63 (LcSOD1), the II values changed from 7.72 (LcSOD3) to 47.31 (LcSOD5), the GRAVY values fluctuated between −0.65 (LcSOD5) to −0.04 (LcSOD6), and the transmembrane domains remained consistently absent. In addition, subcellular localizations of the LcSOD proteins displayed diversity, with four localized in chloroplasts, two in cytoplasm, and one in mitochondrion (Table S3).

Secondary structures of the seven LcSOD proteins encompassed alpha helices, extended strands, beta turns, and random coils. These structures manifested in four distinct patterns: two proteins exhibited alpha helices > random coils > extended strands > beta turns; two proteins displayed random coils > alpha helices > extended strands > beta turns; two proteins presented random coils > extended strands > beta turns > alpha helices; and the remaining one protein featured random coils > extended strands > alpha helices > beta turns (Table S3).

### 3.2. Evolution and Classification of the LcSOD Genes

**Figure 1:**
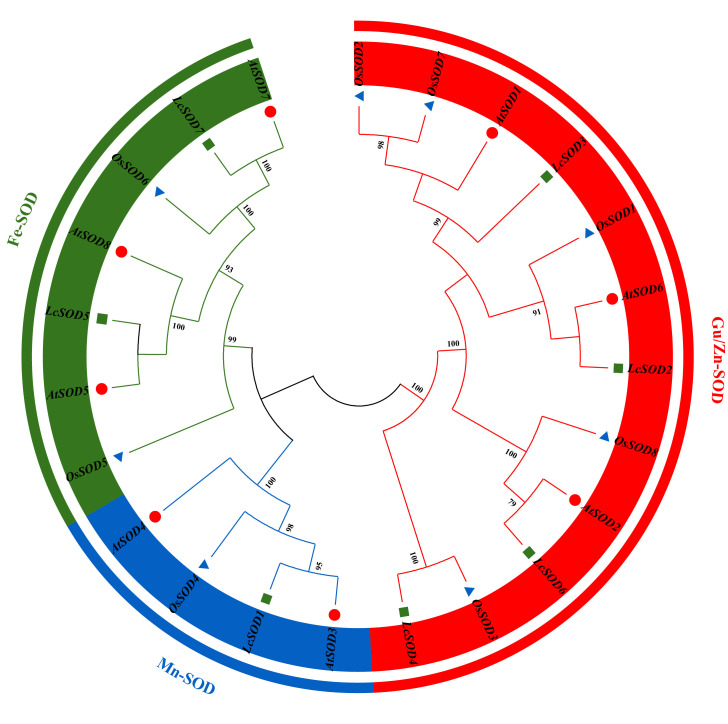
Phylogenetic tree of *SOD* genes from litchi, Arabidopsis, and rice. Green squares represent the *LcSOD* genes, red circles represent *AtSOD* genes, and blue triangles represent *OsSOD* genes.

To investigate the phylogenetic relationships of *SOD* genes between litchi and other plants, a NJ tree was constructed based on *SOD* family groupings from model plants Arabidopsis and rice. The results showed that the LcSOD proteins were clustered into three distinct groups, viz. Cu/Zn-SOD, Fe-SOD, and Mn-SOD. The Cu/Zn-SODs constituted the largest group with four members. In another larger group, Fe-SODs were clustered with Mn-SOD, containing two and one members, respectively. Strikingly, AtSOD proteins possessed a higher presence within the same evolutionary branch as the LcSOD proteins compared to OsSOD proteins (Figure 1).

### 3.3. Chromosomal Distributions and Collinear Relationships of the LcSOD Genes

**Figure 2:**
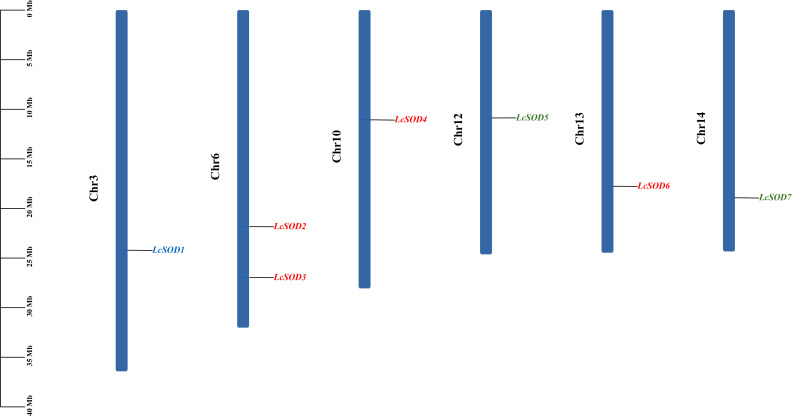
Chromosomal locations of the *LcSOD* gene family members. Gene names in different colors represent distinct groups (similarly hereinafter).

To delineate the distributions of the *LcSOD* genes, a chromosomal location map was constructed. The seven *LcSOD* genes were found to be unevenly distributed across 6 out of the 15 chromosomes (Chr3, Chr6, Chr10, Chr12, Chr13, and Chr14), with genes in the same group scattered randomly. Specifically, Chr6 hosted the most genes, totaling two, followed by Chr3, Chr10, Chr12, Chr13, and Chr14, each containing one gene. Furthermore, the number of genes per chromosome showed no significant positive correlation with chromosome length (Figure 2 and Table S3).

**Figure 3:**
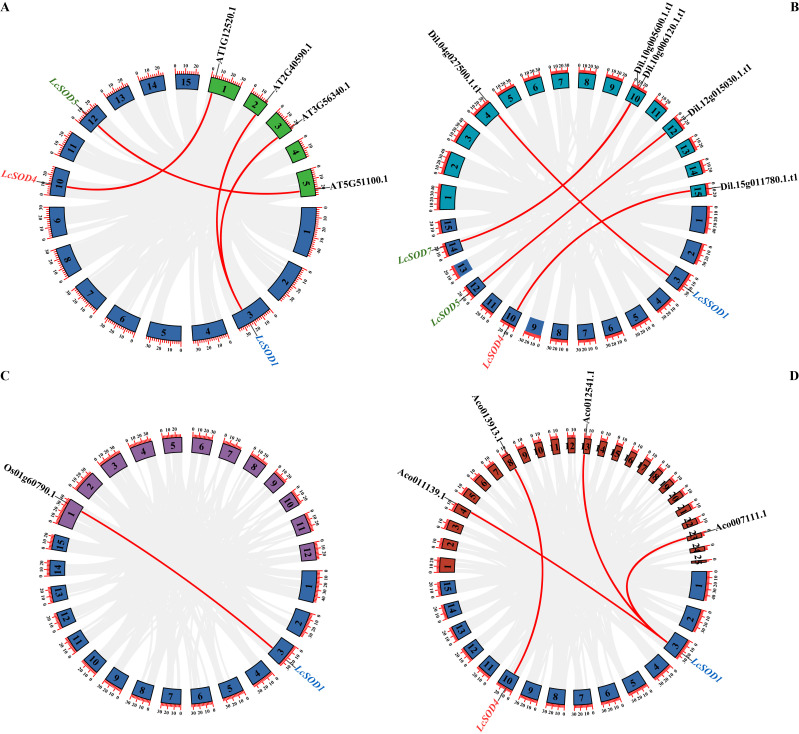
Interspecific collinearity analysis of *SOD* genes between litchi and four species ((**A**) Arabidopsis, (**B**) longan, (**C**) rice, and (**D**) pineapple). Chromosomes in blue belong to litchi, chromosomes in green belong to Arabidopsis, chromosomes in cyan belong to longan, chromosomes in purple belong to rice, and chromosomes in brown belong to pineapple. Gray lines between litchi and other species represent collinear blocks across broad genomic regions, while red lines emphasize the orthologous relationships between *SOD* genes.

To explore the extended evolution of the *LcSOD* gene family, duplication events within the litchi genome and collinearity maps comparing litchi to two dicotyledons (Arabidopsis and longan) and two monocotyledons (rice and pineapple) were elucidated. The results showed no duplication within the *LcSOD* family and closer collinearity between the *LcSOD* genes and dicotyledonous *SOD* genes, particularly longan (five pairs) and Arabidopsis (four pairs), compared to monocotyledonous *SOD* genes (one pair with rice and four pairs with pineapple). Expressly, the three *LcSOD* genes (*LcSOD2*, *LcSOD3*, and *LcSOD6*) lacked collinearity with *SOD* genes in these four species (Figure 3 and Table S4).

### 3.4. Phylogeny, Structures, and Motifs of the LcSOD Genes

**Figure 4:**
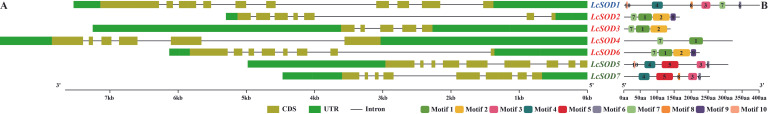
Gene structures and conserved motifs of the *LcSOD* gene family members. (**A**) Exon-intron structures of the *LcSOD* genes. (**B**) Motif patterns of the LcSOD proteins.

To probe the structural divergence of the *LcSOD* genes, their exon-intron architectures were examined. The results indicated that the *LcSOD* genes varied in lengths from 2.95 kb (*LcSOD7*) to 7.66 kb (*LcSOD4*), with intron numbers ranging from four (*LcSOD3*) to nine (*LcSOD1*). Notably, six out of the seven *LcSOD* genes contained untranslated regions (UTRs) at both termini, while *LcSOD5* only had UTRs at one terminus. Additionally, the *LcSOD* genes displayed significant divergence in intron and exon counts across different groups yet maintained relative uniformity within the same group (Figure 4A).

To review the motif configurations of the LcSOD proteins, their motif arrangements were visualized. The results identified 10 conserved motifs (Motif 1–Motif 10) in the LcSOD proteins, each carrying 2–6 motifs with widths ranging from 6 to 50 amino acids. Among them, Motif 1, Motif 2, and Motif 9 were exclusive to Cu/Zn-SODs; Motif 5 was unique to Fe-SODs; Motif 3, Motif 4, Motif 6, Motif 8, and Motif 10 appeared in Fe-SODs and Mn-SOD; and Motif 7 was restricted to Cu/Zn-SODs and Mn-SOD. Obviously, the LcSOD proteins within the same subfamily showed a high degree of conservation in their motif compositions. Further Pfam prediction confirmed that Motif 1 and Motif 2 correspond to Cu/Zn-SOD domain (PF00080), while Motif 3, Motif 4, and Motif 5 correspond to Fe/Mn-SOD domain (PF00081 and PF02777) (Figure 4B and Table S5).

### 3.5. GO Enrichment and Protein-Protein Interaction Network of the LcSOD Genes

**Figure 5:**
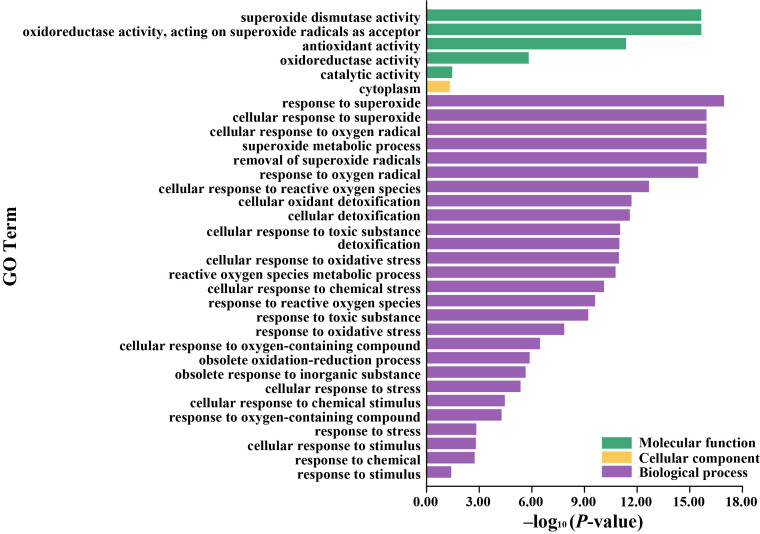
Go annotations of the *LcSOD* gene family members. The extent of GO term enrichment rises as *p*-values diminish.

To uncover functional roles and biological processes linked to the *LcSOD* genes, a GO enrichment analysis was performed. The results indicated that these genes were predominantly enriched in SOD activity, oxidoreductase activity, and antioxidant activity in terms of molecular functions, facilitating the removal of free radicals and safeguarding cells from oxidative damage. Regarding cellular components, the genes primarily functioned in cytoplasm. During biological processes, the genes were implicated in response to superoxide, cellular responses to oxygen radicals, and superoxide metabolic processes, as well as defense and stress responses to chemical and environmental stimuli, highlighting their importance in plant immunity and adaptation mechanisms (Figure 5 and Table S6).

**Figure 6:**
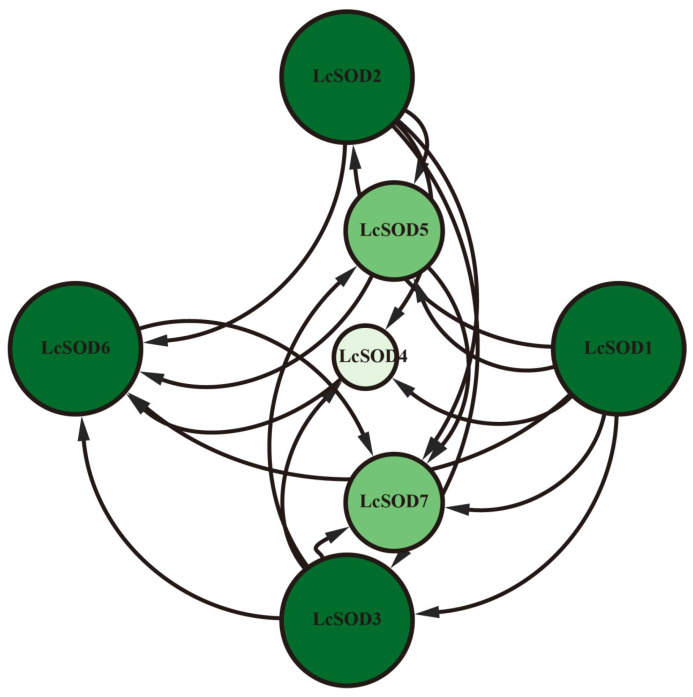
Protein-protein interaction network of the *LcSOD* gene family members. Circle sizes represent network contributions and arrowed lines represent protein interactions.

To assess direct and indirect interactions among the LcSOD proteins, a protein-protein interaction network was established. The analysis unveiled a robust network comprising seven LcSOD proteins and 19 distinct interactions. Among them, LcSOD1, LcSOD2, LcSOD3, and LcSOD6 emerged as key nodes, each engaging with six proteins. LcSOD5 and LcSOD7 followed, interacting with five proteins each, while LcSOD4 exhibited the lowest interaction capacity, connecting with four proteins (Figure 6).

### 3.6. Cis-Acting Elements of the LcSOD Genes

**Figure 7:**
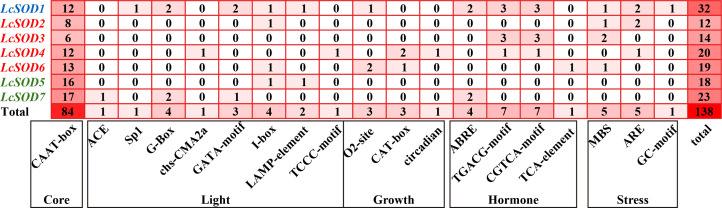
Cis-element distributions in promoters of the *LcSOD* gene family members. The heatmap values represent cis-acting element frequency, with darker colors indicating higher frequencies.

To understand the regulatory mechanisms and functional differentiation of the *LcSOD* genes, an analysis was conducted for their promoter regions. The results showed a total of 138 distinct elements, which were identified and categorized into five functional groups: 84 core elements, 17 light-related elements, 7 growth-related elements, 19 hormone-related elements, and 11 stress-related elements. In detail, core elements were predominant, with 84 enhancer-related elements identified; light-related elements comprised 2 light-responsive and 15 part-of-light-response elements; growth-related elements comprised 3 elements for zein metabolism regulation, 3 for meristem expression, and 1 for circadian control; hormone-related elements consisted of 4 involving abscisic acid, 14 concerning methyl jasmonate, and 1 regarding salicylic acid; and stress-related elements incorporated 5 responsive to drought, 5 inducible by anaerobic conditions, and 1 inducible under anoxic conditions. Distinctly, cis-acting elements in the promoter regions of the *LcSOD* genes within the same group had relatively consistent distribution patterns (Figure 7 and Table S7).

### 3.7. Expression Profiles of the LcSOD Genes in Various Tissues and Their Responses to Abiotic Stresses

**Figure 8:**
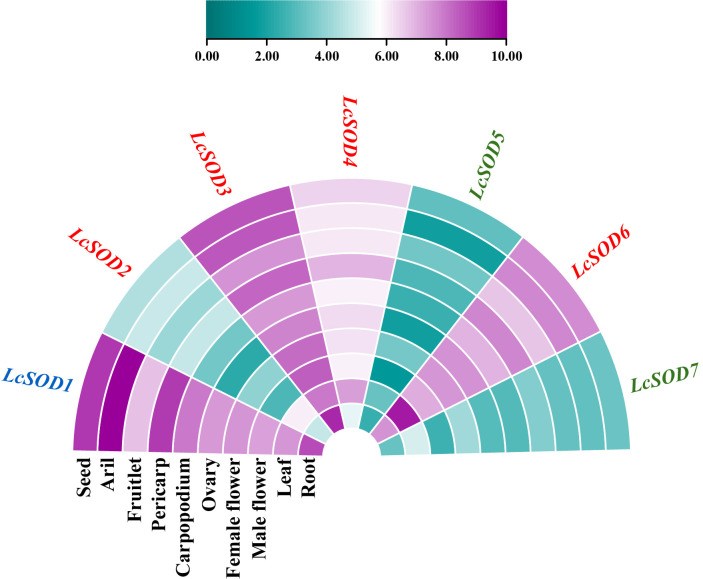
Expression profiles of the *LcSOD* gene family members in different tissues. Purple and cyan represent up- and downregulated expression, respectively; darker shades represent more pronounced expression changes in either direction.

To examine tissue-specific expression and functional divergence of the *LcSOD* genes, we collected their expression data across ten tissues, including roots, leaves, male flowers, female flowers, ovaries, carpopodiums, pericarp, fruitlets, aril, and seeds. The results indicated that all seven *LcSOD* genes were expressed in all tissues but with varying levels, *LcSOD3* had the highest expression in most tissues (excluding leaves, carpopodiums, pericarp, aril, and seeds), *LcSOD5* exhibited the lowest in most tissues (excluding fruitlets), other *LcSOD* genes displayed distinct expression profiles, and the *LcSOD* genes in the same group often had similar expression patterns (Figure 8 and Table S8).

**Figure 9:**
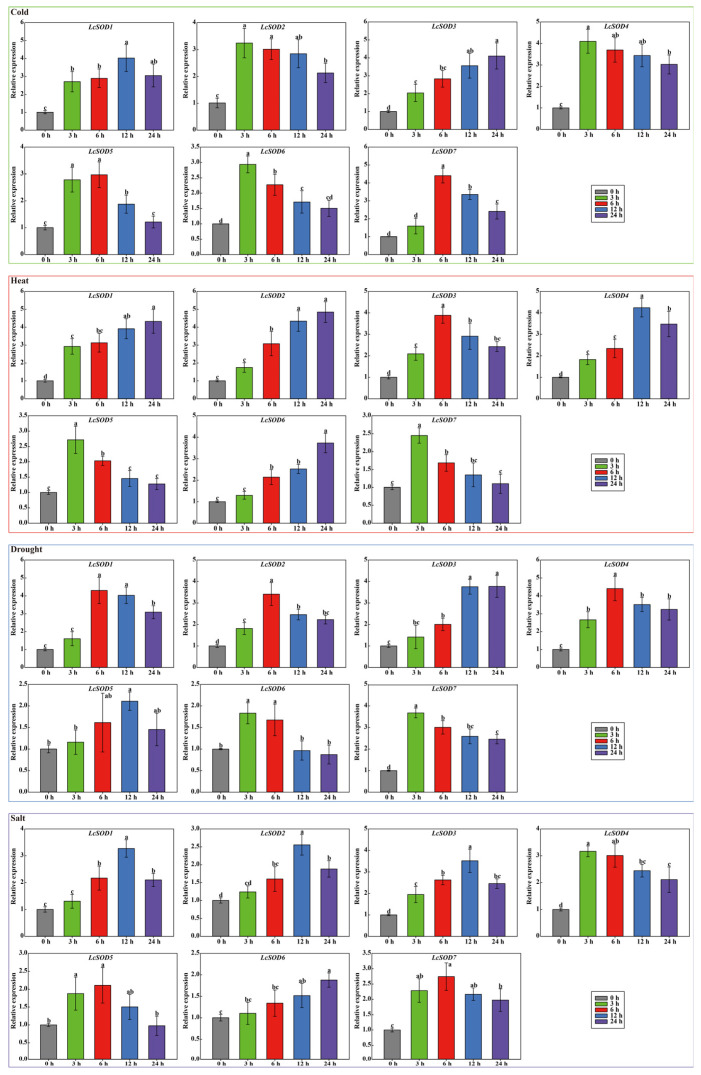
Expression patterns of the *LcSOD* gene family members under various stresses. Error bars represent standard deviations across three replicates, with lowercase letters denoting significant differences (*p* < 0.05) (similarly hereinafter).

To determine the responsiveness of the *LcSOD* genes to diverse stresses, the expression changes of each *LcSOD* gene were measured in leaf tissues. The results showed that the seven *LcSOD* genes displayed varied expressions in leaves under abiotic stresses, yet not all genes exhibited a positive correlation between treatment duration and the intensity of genetic reactions. Specifically, under cold stress, three genes reached peak expression at 3 h, two at 6 h, one at 12 h, and one at 24 h; for heat stress, peak expression occurred at 3 h for two genes, 6 h for one, 12 h for one, and 24 h for three; drought stress induced peak expression at 3 h for two genes, 6 h for three, 12 h for one, and 24 h for one; salt stress prompted peak expression at 3 h for one gene, 6 h for two, 12 h for three, and 24 h for one (Figure 9).

### 3.8. Physiological Responses of Litchi Leaves to Abiotic Stresses

**Figure 10:**
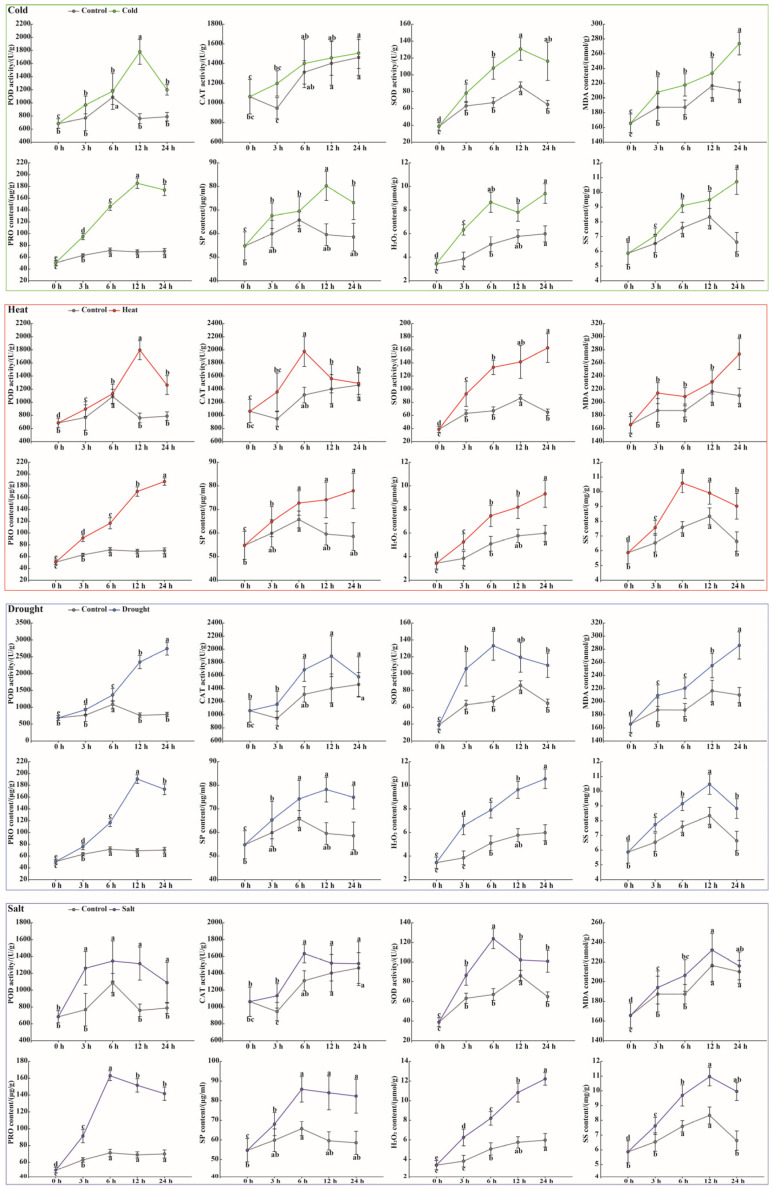
Physiological characteristics of litchi leaves under various stresses.

To quantify and characterize the functional roles of the *LcSOD* genes in conferring stress tolerance, physiological parameters of leaves were assessed under abiotic stresses. The results indicated that all stresses induced significant deviations from control levels, producing distinct temporal signatures. Precisely, cold stress led to a biphasic response: POD, SOD, PRO, and SP peaked at 12 h, whereas CAT, MDA, H_2_O_2_, and SS accumulated maximally at 24 h; heat stress triggered a rapid, acute reaction wherein CAT and SS peaked within 6 h, followed by delayed peaks in POD (12 h) and a suite of other parameters (SOD, MDA, PRO, SP, H_2_O_2_) at 24 h; drought stress prompted an early SOD response at 6 h, with CAT, PRO, SP, and SS maximizing at 12 h; conversely, POD, MDA, and H_2_O_2_ displayed progressive elevation throughout the stress period; salt stress provoked synchronized increases in POD, CAT, SOD, PRO, and SP at 6 h, followed by peaks in MDA and SS at 12 h, whereas H_2_O_2_ content rose continuously (Figure 10).

### 3.9. Correlations Between the LcSOD Gene Expression Levels and Physiological Parameters of Litchi Leaves to Abiotic Stresses

**Figure 11:**
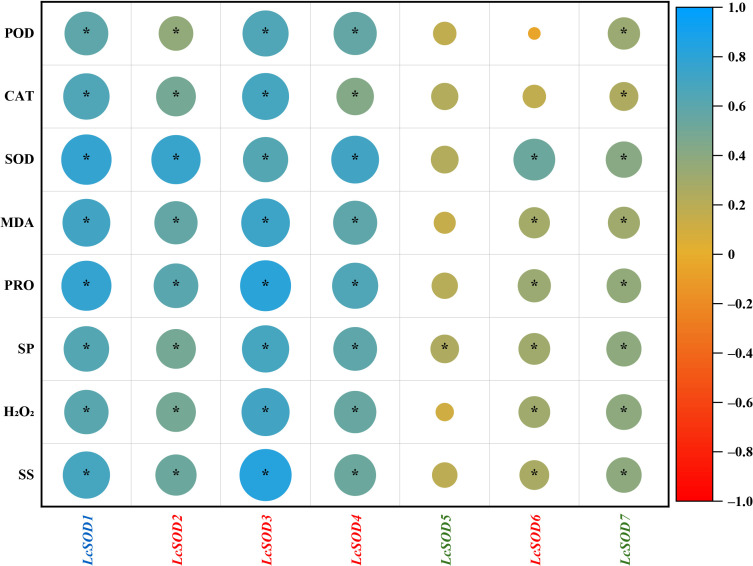
Correlations between the *LcSOD* gene expression and physiological parameters of litchi leaves under various stresses. * represents significant levels at *p* < 0.05.

To dissect the functional relationship between the *LcSOD* gene expression profiles and physiological stress responses, their correlations were executed under abiotic stresses. The results showed significant positive correlations between the *LcSOD* gene expression and physiological parameters: expression of five genes (excluding *LcSOD5* and *LcSOD6*) correlated with POD and CAT activities; six genes (excluding *LcSOD5*) with SOD activity, MDA, PRO, H_2_O_2_, and SS contents; and seven genes with SP content (Figure 11).

## 4. Discussion

SOD is central to plant antioxidant defense, effectively detoxifying ROS in response to environmental stressors [3]. Presently, the *SOD* gene family in litchi remains underexplored and requires comprehensive characterization. Unraveling this family could bridge critical knowledge gaps, offer vital clues for functional genetic research, and facilitate genetic enhancement of stress tolerance in litchi. This study identified seven *SOD* genes from litchi genome, a total that exceeds three in birdsfoot trefoil (*Lotus japonicus* Thunb.) and three in common bean (*Phaseolus vulgaris* Linn.), yet falls below thirteen in wild soybean (*Glycine soja* Sieb. ex Zucc.) and fourteen in cultivated soybean (*Glycine max* (Linn.) Merr.) [23]. The variation in the number of *SOD* family members is likely driven by genome sizes and whole-genome duplication events [24,25]. The seven *LcSOD* genes encoded proteins with diverse lengths and masses, most of which exhibited acidic (pI < 7.00), stable (II < 40.00), and hydrophilic (GRAVY < 0) characteristics (Table S3), consistent with findings in barley (*Hordeum vulgare* Linn.) [26] and Chinese cymbidium (*Cymbidium sinense* Jack. ex Andr.) [27]. Notably, Cu/Zn-SODs (LcSOD2, LcSOD3, LcSOD4, and LcSOD6) and Fe-SODs (LcSOD5 and LcSOD7) were likely expressed in the cytoplasm and chloroplasts, while Mn-SOD (LcSOD1) was localized to mitochondrion, in line with observations of eelgrass (*Zostera marina* L.) [28] and common tobacco (*Nicotiana tabacum* L.) [29]. This distribution enables free radical homeostasis in cells by functioning across multiple compartments. The absence of transmembrane domains in all LcSOD proteins implied that they were not membrane-anchored, supporting their functional collaboration across different organelles.

The *LcSOD* gene family was phylogenetically categorized into three groups, with Cu/Zn-SODs as the most abundant and Mn-SOD the least (Figure 1), aligning with previous research on rapeseed (*Brassica napus* L.) [30] and Chinese cymbidium [27]. On the entire phylogenetic tree, Fe-SODs and Mn-SODs from different plants clustered together and were distinctly separated by a high bootstrap value, indicating their common ancestral origin. This conclusion is supported by findings in upland cotton (*Gossypium hirsutum* L.) [9]. Moreover, the *LcSOD* genes clustered closely with *SOD* genes from Arabidopsis, reflecting evolutionary conservation of the gene family. Furthermore, the *LcSOD* genes displayed significantly higher collinearity with dicotyledonous *SOD* genes than with monocotyledonous ones (Figure 3 and Table S4), perhaps because of the early evolutionary divergence between dicotyledons and monocotyledons within the angiosperm lineage [17]. This finding reinforces the idea that the gene family exhibits significant evolutionary conservation. Visibly, three *LcSOD* genes (*LcSOD2*, *LcSOD3*, and *LcSOD6*) showed no collinearity with *SOD* genes in four species, suggesting that they may have emerged after species divergence.

Structural analysis of the *LcSOD* genes uncovered distinct exon-intron variations (Figure 4A), implying frequent intron addition or loss events in their evolutionary history, where higher intron counts were associated with elevated recombination rates. This pattern aligns with observations in *SOD* genes from foxtail millet (*Setaria italic* L.) [31] and Danshen (*Salvia miltiorrhiza* Bunge) [32]. The *LcSOD* gene family also featured a complex conserved motif composition (Figure 4B). The identification of multiple conserved motifs, especially those unique to specific groups, suggested their critical roles in group-specific regulatory interactions. It is supported by that identified in barrel medic (*Medicago truncatula* Gaertn.) [33] and carrot (*Daucus carota* L.) [5]. Remarkably, these group-specific motifs may function as putative transcription factor binding sites, thereby recruiting distinct regulatory complexes to confer subfunctionalization among the *LcSOD* family members. This hypothesis is substantiated by experimental evidence: *WRKY50*-mediated regulation in rice [34], bZIP binding in cultivated soybean [35], and MYC2 activation in wild tobacco (*Nicotiana attenuate* Torr. ex S. Watson) [36]. As a whole, the *LcSOD* family members displayed intragroup uniformity in gene structures and conserved motifs, with divergence observed among different groups, indicating that genes within the same group have highly conserved structures and functions. This signature is analogous to the results from rubber tree (*Hevea brasiliensis* (Willd. ex A. Juss.) Muell. Arg.) [37] and cassava (*Manihot esculenta* Crantz) [38]. These evolutionary changes likely underpinned the functional diversification of the *LcSOD* genes, shaping their distinct expression profiles and functional roles in stress responses.

Cis-acting elements are pivotal in gene regulatory networks, interacting with transcription factors (TFs) to modulate transcriptional activity in target genes [39]. Here, promoter regions of the *LcSOD* genes were found to contain diverse cis-acting elements related to hormone signaling and stress responses, with at least two such elements present in six of the seven genes (excluding *LcSOD5*) (Figure 7 and Table S7). Crucially, the identified cis-elements exhibited distinct signaling specificities that might orchestrate the *LcSOD*-mediated stress responses through well-defined hormonal pathways, rather than functioning as simple modular accessories. The ABRE (abscisic acid (ABA)-responsive element) serves as a core switch for ABA-dependent signaling, where it is recognized by bZIP-type TFs (e.g., AREB and ABF) to activate gene expression under drought, salinity, and oxidative stress conditions [40]. The enrichment of ABRE in the *LcSOD* promoters suggested that these genes were likely integrated into the ABA-mediated antioxidant defense network, a mechanism widely conserved across upland cotton [9] and Danshen [32]. Meanwhile, the TGACG-motif and CGTCA-motif function as methyl jasmonate (MeJA)-responsive elements that bind to MYC2 and other bHLH-type TFs, thereby activating jasmonic acid (JA) signaling cascades [41]. These elements not only regulate defense gene expression but also modulate ROS homeostasis through cross-talk with ABA pathways, as demonstrated in rapeseed [30] and tea plant (*Camellia sinensis* L.) [42]. The presence of both ABA and MeJA-responsive elements in the *LcSOD* promoters implied a synergistic regulatory module wherein ABA and JA pathways converged to fine-tune SOD activity during complex stress scenarios. Additionally, the TCA-element (salicylic acid-responsive element), MBS (drought-responsive element), ARE (anaerobic induction-responsive element), and GC-motif (anoxic induction-responsive element) further expand this regulatory repertoire, enabling the *LcSOD* genes to respond to multiple abiotic cues through combinatorial control. This multi-hormone responsive architecture provides a mechanistic basis for the precise spatiotemporal expression of the *LcSOD* genes, which is consistent with findings reported for *SOD* genes in foxtail millet [31] and Indian or brown mustard (*Brassica juncea* (L.) Czern.) [10].

Tissue-specific expression of plant genes is key for understanding their functions, as noted by Zhang et al. [43]. This is also true for the *LcSOD* genes (Figure 8 and Table S8), whose varied expression across tissues reflected their diverse roles and supported analyses of phylogenetic relationships, gene structures, conserved motifs, and cis-acting elements. Visibly, high expression (over ten FPKM) of five *LcSOD* genes in roots suggested their role in root growth and stress adaptation; six genes preferential in leaves appeared to be associated with phloem unloading; four genes enriched in flowers and ovaries might regulate flower organ differentiation and development; and five genes were predominantly expressed in carpopodiums, pericarp, fruitlets, aril, and seeds, implying their likely central roles in seed development and fruit ripening. These findings align with prior studies in upland cotton [9] and rapeseed [30]. Lastly, this expression pattern provides a foundation for understanding functional diversification of the *LcSOD* gene family.

The dynamic expression of the *LcSOD* genes under cold, heat, drought, and salt stresses revealed a complex regulatory landscape, with upregulation and downregulation varying by stress type and duration (Figure 9). Early upregulation of certain genes at 3 h likely reflected their role in rapid stress perception and signaling. Later peaks at 6 h and 12 h suggested their potential involvement in sustained stress responses and recovery processes. Final peaks at 24 h implied ongoing stress responses, while downregulation observed at specific times relative to 0 h illustrated potential negative regulatory roles or feedback mechanisms that regulated stress responses and mitigated excessive damage in litchi. *SOD* genes in banana [13] and carrot [5] showed similar expression profiles, highlighting their diverse and specific roles in response to abiotic stresses. Later on, physiological analysis of litchi leaves under abiotic stresses demonstrated notable elevation in antioxidant enzyme activities (POD, CAT, and SOD) and metabolite contents (MDA, PRO, SP, H_2_O_2_, and SS) (Figure 10), closely matching the expression patterns of the *LcSOD* genes. This positive correlation inferred that the *LcSOD* genes likely perform a critical role in coordinating molecular and physiological responses of litchi to environmental stresses (Figure 11). These results not only validate the role of *LcSOD* genes in abiotic stress tolerance but also help identify promising candidates for enhancing genetic resilience in litchi.

## 5. Conclusions

This study identified seven *LcSOD* genes distributed across six chromosomes for the first time from the litchi genome, classifying them into three groups with highly conserved structural and motif distributions. The *LcSOD* gene family exhibited greater homology with dicotyledonous *SOD* genes than with monocotyledonous ones. Importantly, GO annotations, cis-acting elements, tissue-specific expression, and responses of the *LcSOD* genes to abiotic stresses collectively highlighted their critical regulatory functions in growth, development, and stress tolerance. Overall, comprehensive analysis of the *LcSOD* genes provides valuable insights into their roles in antioxidant defense and stress adaptation. These findings not only enrich our understanding of *SOD* gene family in subtropical fruit trees but also offer a basis for future functional studies aimed at improving stress resistance in litchi and related species.

## Data Availability

The original contributions presented in this study are included in the article/Supplementary Material. Further inquiries can be directed to the corresponding author.

## Supplementary Materials

The following supporting information can be downloaded at https://www.mdpi.com/article/10.3390/antiox15010014/s1. Table S1: Amino acid sequences of *SOD* genes derived from litchi, Arabidopsis, and rice; Table S2: The primer list used for qRT-PCR; Table S3: Basic characteristics of the *LcSOD* genes; Table S4: Orthologous gene pairs of *SOD* genes between litchi and Arabidopsis/longan/rice/pineapple; Table S5: Conservative motif information of the *LcSOD* genes; Table S6: GO annotation analysis of the *LcSOD* genes; Table S7: Cis-acting elements present in promoter regions of the *LcSOD* genes; Table S8: RNA-seq data for expression of the *LcSOD* genes across various tissues.

## References

[1] Wang P., Zhu J.K. (2025). Plant water stress sensing: Osmosensors start to make sense. Sci. Bull..

[2] Gangwar R., Kumari P., Chatrath A., Prasad R. (2020). Characterisation of recombinant thermostable manganese-superoxide dismutase (*NeMnSOD*) from *Nerium oleander*. Mol. Biol. Rep..

[3] Mansoor S., Wani O.A., Lone J.K., Manhas S., Kour N., Alam P., Ahmad A., Ahmad P. (2022). Reactive oxygen species in plants: From source to sink. Antioxidants.

[4] Soares C., Carvalho M.E.A., Azevedo R.A., Fidalgo F. (2018). Plants facing oxidative challenges—A little help from the antioxidant networks. Environ. Exp. Bot..

[5] Zameer R., Fatima K., Azeem F., ALgwaiz H.I.M., Sadaqat M., Rasheed A., Batool R., Shah A.N., Zaynab M., Shah A.A. (2022). Genome-wide characterization of superoxide dismutase (SOD) genes in *Daucus carota*: Novel insights into structure, expression, and binding interaction with hydrogen peroxide (H_2_O_2_) under abiotic stress condition. Front. Plant Sci..

[6] Jiang W., Yang L., He Y., Zhang H., Li W., Chen H., Ma D., Yin J. (2019). Genome-wide identification and transcriptional expression analysis of superoxide dismutase (SOD) family in wheat (*Triticum aestivum*). Peer J..

[7] Lin Y.-L., Lai Z.-X. (2013). Superoxide dismutase multigene family in longan somatic embryos: A comparison of CuZn-SOD, Fe-SOD, and Mn-SOD gene structure, splicing, phylogeny, and expression. Mol. Breed..

[8] Sheng Y., Abreu I.A., Cabelli D.E., Maroney M.J., Miller A.-F., Teixeira M., Valentine J.S. (2014). Superoxide dismutases and superoxide reductases. Chem. Rev..

[9] Wang W., Zhang X., Deng F., Yuan R., Shen F. (2017). Genome-wide characterization and expression analyses of superoxide dismutase (*SOD*) genes in *Gossypium hirsutum*. BMC Genom..

[10] Verma D., Lakhanpal N., Singh K. (2019). Genome-wide identification and characterization of abiotic-stress responsive *SOD* (*superoxide dismutase*) gene family in *Brassica juncea* and *B. rapa*. BMC Genom..

[11] Kliebenstein D.J., Monde R.A., Last R.L. (1998). Superoxide dismutase in Arabidopsis: An eclectic enzyme family with disparate regulation and protein localization. Plant Physiol..

[12] Shiraya T., Mori T., Maruyama T., Sasaki M., Takamatsu T., Oikawa K., Itoh K., Kaneko K., Ichikawa H., Mitsui T. (2015). Golgi/plastid-type manganese superoxide dismutase involved in heat-stress tolerance during grain filling of rice. Plant Biotechnol. J..

[13] Feng X., Lai Z., Lin Y., Lai G., Lian C. (2015). Genome-wide identification and characterization of the superoxide dismutase gene family in *Musa acuminate* cv. *Tianbaojiao* (AAA group). BMC Genom..

[14] Kaouthar F., Ameny F.K., Yosra K., Walid S., Ali G., Faiçal B. (2016). Responses of transgenic *Arabidopsis* plants and recombinant yeast cells expressing a novel durum wheat manganese superoxide dismutase *TdMnSOD* to various abiotic stresses. J. Plant Physiol..

[15] Chen Y., Wu H., Hao Z., Zhu L., Lu L., Shi J., Chen J. (2023). The identification and expression analysis of the *Liriodendron chinense* (Hemsl.) Sarg. *SOD* gene family. Forests.

[16] Chen X., Li D., Guo J., Wang Q., Zhang K., Wang X., Shao L., Luo C., Xia Y., Zhang J. (2024). Identification and analysis of the superoxide dismutase (SOD) gene family and potential roles in high-temperature stress response of herbaceous peony (*Paeonia lactiflora* Pall.). Antioxidants.

[17] Yang J., Chen R., Liu W., Fan C. (2025). Genome-wide identification, phylogenetic investigation and abiotic stress responses analysis of the *PP2C* gene family in litchi (*Litchi chinensis* Sonn.). Front. Plant Sci..

[18] Yang J., Chen R., Xiang X., Liu W., Fan C. (2024). Genome-wide identification and expression analysis of the class III peroxidase gene family under abiotic stresses in litchi (*Litchi chinensis* Sonn.). Int. J. Mol. Sci..

[19] Li J., Chen C., Zeng Z., Wu F., Feng J., Liu B., Mai Y., Chu X., Wei W., Li X. (2024). SapBase: A central portal for functional and comparative genomics of Sapindaceae species. J. Integr. Plant Biol..

[20] Chen C., Wu Y., Li J., Wang X., Zeng Z., Xu J., Liu Y., Feng J., Chen H., He Y. (2023). TBtools-II: A “one for all, all for one” bioinformatics platform for biological big-data mining. Mol. Plant.

[21] Livak K.J., Schmittgen T.D. (2001). Analysis of relative gene expression data using real time quantitative PCR and the 2^−ΔΔCT^ method. Methods.

[22] Wei Y.Z., Hu F.C., Hu G.B., Li X.J., Huang X.M., Wang H.C. (2011). Differential expression of anthocyanin biosynthetic genes in relation to anthocyanin accumulation in the pericarp of *Litchi Chinensis* Sonn. PLoS ONE.

[23] Aleem M., Aleem S., Sharif I., Wu Z., Aleem M., Tahir A., Atif R.M., Cheema H.M.N., Shakeel A., Lei S. (2022). Characterization of *SOD* and *GPX* gene families in the soybeans in response to drought and salinity stresses. Antioxidants.

[24] Liu Y., Liu X., Yang D., Yin Z., Jiang Y., Ling H., Huang N., Zhang D., Wu J., Liu L. (2022). A comprehensive identification and expression analysis of VQ motif-containing proteins in sugarcane (*Saccharum spontaneum* L.) under phytohormone treatment and cold stress. Int. J. Mol. Sci..

[25] Zhang G., Wang F., Li J., Ding Q., Zhang Y., Li H., Zhang J., Gao J. (2015). Genome-Wide Identification and analysis of the VQ motif-containing protein family in Chinese cabbage (*Brassica rapa* L. ssp. *Pekinensis*). Int. J. Mol. Sci..

[26] Zhang X., Zhang L., Chen Y., Wang S., Fang Y., Zhang X., Wu Y., Xue D. (2021). Genome-wide identification of the SOD gene family and expression analysis under drought and salt stress in barley. Plant Growth Regul..

[27] Li R., Lin S., Yan Y., Chen Y., Wang L., Zhou Y., Tang S., Liu N. (2025). Genome-wide identification of superoxide dismutase (*SOD*) gene family in *Cymbidium* species and functional analysis of *CsSODs* under salt stress in *Cymbidium sinense*. Horticulturae.

[28] Zang Y., Chen J., Li R., Shang S., Tang X. (2020). Genome-wide analysis of the superoxide dismutase (SOD) gene family in *Zostera marina* and expression profile analysis under temperature stress. PeerJ.

[29] Huo C., He L., Yu T., Ji X., Li R., Zhu S., Zhang F., Xie H., Liu W. (2022). The superoxide dismutase gene family in *Nicotiana tabacum*: Genome-wide identification, characterization, expression profiling and functional analysis in response to heavy metal stress. Front. Plant Sci..

[30] Su W., Raza A., Gao A., Jia Z., Zhang Y., Hussain M.A., Mehmood S.S., Cheng Y., Lv Y., Zou X. (2021). Genome-wide analysis and expression profile of superoxide dismutase (SOD) gene family in rapeseed (*Brassica napus* L.) under different hormones and abiotic stress conditions. Antioxidants.

[31] Wang T., Song H., Zhang B., Lu Q., Liu Z., Zhang S., Guo R., Wang C., Zhao Z., Liu J. (2018). Genome-wide identification, characterization, and expression analysis of superoxide dismutase (SOD) genes in foxtail millet (*Setaria italica* L.). 3 Biotech.

[32] Han L.M., Hua W.P., Cao X.Y., Yan J.A., Chen C., Wang Z.Z. (2020). Genome-wide identification and expression analysis of the *superoxide dismutase* (*SOD*) gene family in *Salvia miltiorrhiza*. Gene.

[33] Song J., Zeng L., Chen R., Wang Y., Zhou Y. (2018). In silico identification and expression analysis of superoxide dismutase (SOD) gene family in *Medicago truncatula*. 3 Biotech.

[34] Huang S., Hu L., Zhang S., Zhang M., Jiang W., Wu T., Du X. (2021). Rice OsWRKY50 mediates ABA-dependent seed germination and seedling growth, and ABA-independent salt stress tolerance. Int. J. Mol. Sci..

[35] Chai M., Fan R., Huang Y., Jiang X., Wai M.H., Yang Q., Su H., Liu K., Ma S., Chen Z. (2022). *GmbZIP152*, a soybean bZIP transcription factor, confers multiple biotic and abiotic stress responses in plant. Int. J. Mol. Sci..

[36] Choung S., Lee G., Kang M., Park K., Park E., Lee S., Song J., Goldberg J.K., Baldwin I.T., Joo Y. (2025). *MYC2* and *MYC3* orchestrate pith lignification to defend *Nicotiana attenuata* stems against a stem-boring weevil. New Phytol..

[37] Yu W., Kong G., Chao J., Yin T., Tian H., Ya H., He L., Zhang H. (2022). Genome-wide identification of the rubber tree superoxide dismutase (*SOD*) gene family and analysis of its expression under abiotic stress. PeerJ.

[38] Zheng L., Hamidou A.A., Zhao X., Ouyang Z., Lin H., Li J., Zhang X., Luo K., Chen Y. (2023). Superoxide dismutase gene family in cassava revealed their involvement in environmental stress via genome-wide analysis. iScience.

[39] Zhao Y., Zhao H., Wang Y., Zhang X., Zhao X., Yuan Z. (2020). Genome-wide identification and expression analysis of MIKC-type MADS-box gene family in *Punica granatum* L.. Agronomy.

[40] Wang H., Zi Y., Rong X., Zhang Q., Nie L., Wang J., Ren H., Zhang H., Liu X. (2025). Expression analysis of the ABF gene family in *Actinidia chinensis* under drought stress and the response mechanism to abscisic acid. Horticulturae.

[41] Durand A.N., Pauwels L., Goossens A. (2016). The ubiquitin system and jasmonate signaling. Plant.

[42] Zhou C., Zhu C., Fu H., Li X., Chen L., Lin Y., Lai Z., Guo Y. (2019). Genome-wide investigation of *superoxide dismutase* (*SOD*) gene family and their regulatory miRNAs reveal the involvement in abiotic stress and hormone response in tea plant (*Camellia sinensis*). PLoS ONE.

[43] Zhang L.H., Zhu L.C., Xu Y., Lü L., Li X.G., Li W.H., Liu W.D., Ma F.W., Li M.J., Han D.G. (2023). Genome-wide identification and function analysis of the sucrose phosphate synthase *MdSPS* gene family in apple. J. Integr. Agr..

